# Neuroprotective Effects of Geniposide in SH-SY5Y Cells and Primary Hippocampal Neurons Exposed to A*β*42

**DOI:** 10.1155/2014/284314

**Published:** 2014-11-18

**Authors:** Ping Sun, Haimin Ding, Mi Liang, Xiaojing Li, Weichuan Mo, Xu Wang, Ying Liu, Rongqiao He, Qian Hua

**Affiliations:** ^1^School of Preclinical Medicine, Beijing University of Chinese Medicine, 11 Bei San Huan Dong Road, Chaoyang District, Beijing 100029, China; ^2^State Key Laboratory of Brain and Cognitive Science, Institute of Biophysics, Chinese Academy of Sciences, 15 Datun Road, Chaoyang District, Beijing 100101, China

## Abstract

Our former studies have suggested that TongLuoJiuNao (TLJN) is clinically efficacious in the treatment of dementia and improving learning and memory in AD models. When A*β* aggregated with oligomer, it is known to be able to induce cellular toxicity as well as cognitive impairment. We tested the possibility that TLJN affects the formation of A*β* oligomers. In our experiment, TLJN improved cell viability, inhibited LDH release, and promoted the outgrowth of neurites of neurons treated with A*β*. Geniposide, the main component of TLJN, could increase the cell viability of SY5Y-APP695sw cells. The cytotoxicity of pretreated A*β* with geniposide was decreased in a dose-dependent manner. SDS-PAGE and Western blotting showed that geniposide and TLJN stimulated A*β* oligomer assembly. Compared with the control, more and longer fibrils of A*β* in the presence of geniposide were observed under electron microscope though the fibrils became less sensitive to thioflavin T staining. In sum, geniposide is able to protect neurons from A*β*-induced damage by remodeling A*β*.

## 1. Introduction

Alzheimer's disease (AD) is the most prevalent neurodegenerative disease of the brain, characterized clinically by progressive decline of intellectual abilities, including memory and reasoning, and the irreversible loss of neurons [1, 2]. In AD, large numbers of extracellular amyloid plaques and intracellular neurofibrillary tau-tangles are found in cerebral cortex and hippocampus area. The main component of these plaques is *β*-amyloid (A*β*) [3].

Recent evidence shows that accumulation of A*β* as soluble extracellular high molecular weight (HMW) oligomeric A*β* species, rather than deposition of amyloid plaques, may be specifically related to spatial memory deficits in AD, including inhibition of hippocampal long-term potentiation, disruption of synaptic plasticity, and causal acceleration of early-onset behavioral impairment [4]. A*β* assembly is an intricate process which is considerably more complex than a simple conversion of soluble monomer to oligomer to fibril. There are a variety of different aggregate morphologies, including soluble oligomers, protofibrils, diffuse plaques, and fibrillar deposits seen in the senile plaques [5]. All of these aggregated forms seem to be dominated by the so-called *β*-sheet structure [5]. The most crucial factor determining A*β* toxicity is the aggregation state. Consequently, compounds that inhibit A*β* aggregation, fibrillation, or plaque formation may be capable of protecting neurons from A*β* toxicity and thus display therapeutic potential for the disease [6].

There are several groups focusing on the inhibition of A*β* aggregation and neurotoxicity for the treatment of Alzheimer's disease. Tjernberg and some other groups developed A*β* aggregation inhibitors which targeted the peptide sequence itself by using short peptide fragments homologous to the full-length wild-type protein and they could inhibit aggregation of the amyloidogenic protein A*β* and A*β*-induced pathological changes [7–9]. Besides these small molecule inhibitors, natural products are also widely studied in the inhibition of A*β* aggregation. Experimental and epidemiological evidence suggests that natural polyphenolic compounds, such as those found in teas, berries, fruits, spices, and plants, have antiaggregate properties [10]. Curcumin, (-)epigallocatechin-3-gallate (EGCG), and Ginkgo biloba could distort the normal aggregation of A*β* and protect neuron from A*β* oligomers [5, 11, 12].

Herbal remedies have a long history in treating various symptoms of many diseases, such as stroke and Alzheimer's disease [13]. TongLuoJiuNao (TLJN), which is prepared according to a traditional Chinese medicine (TCM) formula, is clinically efficacious in the treatment of ischemic cerebral stroke and dementia (Chinese SFDA: 2004L01620). Previous studies showed that TLJN could protect brain tissue against ischemia after the induction of middle cerebral artery occlusion (MCAO) in rats [14] and improve learning and memory ability by promoting the expression of degrading enzyme of A*β* and clearing amyloid plaques from the AD rat brain [15]. We recently reported that TLJN significantly decreased A*β* production and deposition in the brain of APP23 mice [16]. HPLC detection demonstrated that major components of TLJN are ginsenoside Rg1 and geniposide. Ran et al. had reported that both of ginsenoside Rg1 and geniposide have neuroprotective effects in culture cell of mouse [17]. Mesencephalic dopaminergic cells stressed with glutamate benefit from ginsenoside Rg1 [18], while geniposide can protect PC12 cells from hydrogen peroxide-induced cell death via involvement in the PI3K signaling pathway and activation of the glucagon-like peptide 1 receptor (GLP-1R) [19], as well as rescue formaldehyde induced apoptosis in N2a neuroblastoma cells [20]. Geniposide is also able to regulate insulin secretion through activating GLP-1R in rat INS-1 insulinoma cells [21]. However, neither of the two main components of TLJN belongs to polyphenolic compounds, and whether TLJN have any influence on the formation and metabolism of A*β* fibrils has not yet been investigated. In the present study, we examined the effects of TLJN and its main components on the formation of A*β* aggregates by using fluorescence spectroscopy with thioflavin T (ThT), transmission electron microscope, and electrophoresis.

## 2. Materials and Methods

### 2.1. Materials

TLJN was provided by Kangyuan Pharmaceutical Engineering Limited Company (Beijing, China), and its main ingredients are geniposide and ginsenoside Rg1. The concentrations of geniposide (4.95 mg/mL) and ginsenoside Rg1 (1.02 mg/mL) in TLJN were determined by high performance liquid chromatography (HPLC) [14]. Geniposide and ginsenoside Rg1 standard were purchased from National Institutes for Food and Drug Control (Beijing, China). A*β* was bought from American Peptide Company (Sunnyvale, CA, USA). Cell Counting Kit-8 was obtained from Dojindo (Kumamoto, Japan) and LDH Cytotoxicity Assay Kit was purchased from Cayman (Ann Arbor, MI, USA). Neurobasal, F12 culture medium, trypsin, B27 supplement, and Gluta-MAX-I Supplement were bought from Invitrogen (Carlsbad, CA, USA). Enhanced chemiluminescence (ECL) substrate was obtained from Pierce (Boston, MA, USA). Polyvinylidene difluoride (PVDF) membrane was bought from Millipore (Billerica, MA, USA). Thioflavin T and hexafluoroisopropanol were obtained from Sigma (St. Louis, MO, USA).

### 2.2. A*β* Preparation

One mg A*β*42 was dissolved in 222 *μ*L hexafluoroisopropanol overnight and then lyophilized. The dried extract was then dissolved in DMSO at 5 mM as stock solutions. For kinetic aggregation experiments, stock solutions were further diluted with SP buffer (100 mM sodium phosphate, pH 7.4, 10 mM NaCl) to 20 *μ*M as the final concentration. Then it was incubated at 37°C with or without different tested drugs for 72 h. For cytotoxicity experiments, stock solutions were diluted into F12 culture medium to 500 *μ*M and then incubated at 37°C for 24 h with different treatments.

### 2.3. Cell Culture

 Hippocampal neuronal cultures were obtained from embryonic rat at days 16–18 of gestation as previously described with modification [22]. Briefly, after being dissected, hippocampus was incubated with 0.25% trypsin for 15 minutes at 37°C. Then the cells were shaken and the cell suspension was passed through a 100 *μ*m cell strainer. Neurons were grown in neural basal medium containing 0.5 mM Gluta-MAX and half of the medium was replaced twice a week. Neurons were seeded onto tissue culture dishes and maintained in a humidified incubator with 5% CO_2_ at 37°C. After 7 days' incubation in 24-well culture plates, the hippocampal neurons were used for the experiments described below. SH-SY5Y cells were grown in Dulbecco's Modified Eagle Medium (DMEM, USA) containing 10% fetal bovine serum in 5% CO_2_/90% humid air at 37°C. These cells were plated onto 96-well culture plates at a density of 1 × 10^4^ cells/well for cytotoxicity assays. After 24 h preincubation, the culture medium was replaced with serum-free DMEM supplemented with 10 *μ*M A*β* and different concentrations of drugs. SY5Y-APP695swe cell line stably transfected with the human Swedish mutated APP was used in the experiment when the cells were growing in logarithmic growth phase. Amyloid *β* was overexpressed in SY5Y-APP695swe cells which had cytotoxicity and inhibited the activity of cells. It can be used as an endogenous A*β* toxicity cell model instead of incubating cells with exogenous A*β*. The treatment of SY5Y-APP695swe cell line was similar to SH-SY5Y, while the final culture medium did not include A*β*. TLJN preparation is clinically efficacious in the treatment of ischemic cerebral stroke and dementia, in which ginsenoside Rg1 and geniposide are two major components in the elution through HPLC [14]. Previously, we identified that TLJN has neuroprotective effects on ischemia/reperfusion neurons and brain microvascular endothelial cells [23]. In this work, we attempt to investigate whether TLJN has neuroprotective effects on A*β*-injured neural cells. A*β* stock solutions were diluted into F12 culture medium to 500 *μ*M and then incubated at 37°C for 24 h to facilitate A*β* aggregation. Hippocampal neuronal cultures (inoculation size: 1 × 10^4^) were inoculated in the presence of 10 *μ*M preincubated A*β* supplemented with or without TLJN.

### 2.4. Cytotoxicity Assay

The viability of hippocampal neurons and SH-SY5Y cells was determined by measuring the activity of the lactate dehydrogenase (LDH) released into the culture media with the LDH Assay Kit according to the manufacturer's protocol. The absorbance at 490 nm was measured with a microplate reader and the reference wavelength was 630 nm. The viability of SY5Y-APP695swe cells was evaluated by Cell Counting Kit-8 (CCK-8). Briefly, add 10 *μ*L of the CCK-8 solution and 90 *μ*L of DMEM−/− to each well of the 96-well plate and then incubate the plate for 4 h in the incubator. The absorbance at 450 nm was measured with a microplate reader and the reference wavelength is 630 nm.

### 2.5. Immunohistology

Cell death was also determined by MAP2 antibody (Abcam, Cambridge, MA, USA) and Hoechst 33258 (Sigma, St. Louis, MO, USA) double fluorescent staining. In these cases, hippocampal neurons were cultured at a density of 10^5^ cells/mm^2^ on 8 mm glass coverslips in 24-well plates. After the indicated treatments and fixing by 4% paraformaldehyde, the cells were stained with MAP2 antibody and Hoechst 33258. For each coverslip, ten visual fields were selected randomly using the ANDOR image analysis system (Cold Spring Corporation, NY, USA). The results were expressed as the percentages of death cells.

### 2.6. Analyses of Cell Viability

Cell death was determined by propidium iodide (PI, Sigma, St. Louis, MO) and Hoechst 33258 (Sigma) double fluorescent staining. In these cases, hippocampal neurons were cultured at a density of 10^5^ cells/mm^2^ on 8 mm glass coverslips in 24-well plates. After the indicated treatments, the cells were stained with PI (10 *μ*g/mL) and Hoechst 33258 (10 *μ*g/mL) for 30 min and then fixed by 4% paraformaldehyde. For each coverslip, ten visual fields were selected randomly using the ANDOR image analysis system (Cold Spring Corporation, NY, USA). The results were expressed as the percentages of death cells.

### 2.7. Analysis of Neurites Outgrowth

Cultured rat hippocampal neurons were plated at 35 mm dish with a density of 2 × 10^5^ cells/well. After 3 days' incubation with TLJN, neurons were transfected with 2.5 *μ*g of DNA/dish which encoded recombinant green fluorescent protein (GFP) by ways of Lipofectamine 2000 (Invitrogen, Carlsbad, CA, USA) according to the manufacturer's protocol. Three days after transfection, the morphology of the neurons was observed using a Nikon fluorescence microscope. The length and number of neurons were measured with Image-Pro-Plus 5.0 software (Media Cybernetics, Rockville, MD, USA). A total of 12 wells were measured for each group at each time point. Five sample locations from each well were pictured and examined.

### 2.8. EM Assay

A*β*42 peptide was dissolved in 0.1 M phosphate buffer (pH 7.2), which was incubated in the presence and absence of geniposide at 37°C. The final concentration for both A*β*42 and geniposide was 100 *μ*M. After 24 h incubation, aliquots (5 *μ*L) were placed on carbon-coated copper/rhodium grid. After 1 min, the grid was washed with water and negatively stained with 2% uranyl acetate solution for 1 min. After draining off the excess of staining solution by means of a filter paper, the specimen was transferred for examination in a transmission electron microscope (Tecnai Spirit 120 kV, Hong Kong).

### 2.9. ThT Fluorescence

Ten *μ*L of sample was added to 190 *μ*L of ThT dissolved in 10 mM phosphate buffer, pH 7.4, and then the mixture was vortexed briefly. Fluorescence was determined three times at intervals of 10 s using a Tecan Safire 2 fluorometer. Excitation and emission wavelengths were 450 nm and 482 nm, respectively. Sample fluorescence was determined by averaging the three readings and subtracting the fluorescence of a ThT blank.

### 2.10. SDS-PAGE Staining and Western Blotting

A*β* stock solution was diluted to 100 *μ*M with F12 culture medium with or without TLJN solutions. 20 mL of samples was electrophoresed on a Tricine gel and visualized by silver staining and nonaggregated samples were used as controls in each experiment. Western blotting was carried out as previously described [5]. Amyloid *β* stocking solution was diluted to 40 *μ*M with F12 culture medium with or without geniposide solutions. 20 *μ*L of samples was electrophoresed on a 4–20% gradient Tricine gel (Thermo, Boston, MA, USA). The separated proteins were transferred to a PVDF membrane and the membrane was hybridized with A*β* antibody (1 : 1000 dilution, Cell Signaling Technology, Waltham, USA). HRP-conjugated anti-rabbit/mouse IgG was used as the secondary antibody (1 : 5000 dilution).

### 2.11. Statistical Analysis

Each experiment was repeated for at least three times and results were expressed as mean ± standard deviation (SD) and one way variance analysis followed by Fisher's least significant difference (LSD) test was carried out using SPSS 11.0. Values of *P* < 0.05 were considered statistically significant.

## 3. Results

### 3.1. A Decrease in Cytotoxicity of A*β* on Rat Hippocampal Neurons in the Presence of TLJN

**Figure 1:**
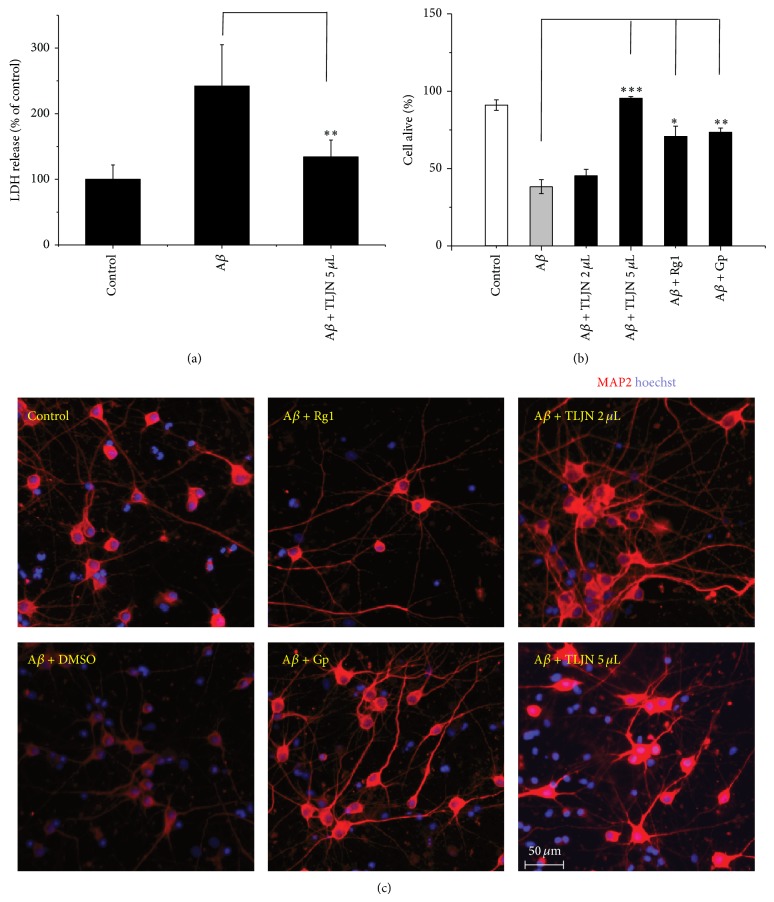
A decrease in cytotoxicity of A*β* on rat hippocampal neurons in the presence of TLJN and its ingredients. Rat hippocampal neurons were cultured with the mixture of TLJN (high dose of TLJN: 5 *μ*L TLJN per mL culture media (containing 83.5 *μ*M geniposide and 8.35 *μ*M ginsenoside Rg1); low dose of TLJN: 2 *μ*L TLJN per mL culture media (containing 25.4 *μ*M geniposide and 2.54 *μ*M ginsenoside Rg1)) or its ingredient (geniposide (83.5 *μ*M) and ginsenoside Rg1 (8.35 *μ*M)) and A*β* (10 *μ*M) for three days. The activities of LDH released in medium (a) were measured after the treatment. The survival rate of neurons was determined by immunohistochemical double staining with PI and Hoechst (b). The morphology of neurons stained with MAP2 and Hoechst were observed (c); bar = 50 *μ*m. Data were represented as mean ± standard deviation (SD); ^*^ *P* < 0.05, ^**^ *P* < 0.01, and ^***^ *P* < 0.001.

To assess neuronal viabilities in the presence of TLJN, the activity of LDH released from neurons was detected by LDH Assay Kit to test whether the cell viability was changed. As shown in Figure 1(a), the LDH activity increased by 142% in medium where the cells were cultured with A*β* compared with those without A*β* treatment as control, indicating the treatment with A*β* leads to the cell death. However, when neurons were treated by supplement of TLJN, the LDH activity significantly decreased to 51%, which decreased by 45% compared with A*β* alone treated group (*P* < 0.05).

To further demonstrate the changes in cell viability, the hippocampal neurons were double stained with Hoechst 33258 and PI antibody to distinguish the alive and dead cells (Figure 1(b)). After the cells were cultured with preincubated A*β*, 38% of the cells were survived which were decreased by 58% compared with control group (Figure 1(b)). When neurons were treated by TLJN of high dose, the survived cells were increased by 148% (*P* < 0.001) compared with A*β* treated alone group. Besides, MAP2 expressions were stronger in TLJN treatment groups compared to A*β* treated alone group (Figure 1(c)).

In fact, geniposide and ginsenoside Rg1 are main ingredients of TLJN; thus we detected the percentages of alive cells after being treated with geniposide and ginsenoside Rg1. Geniposide increased the percentages of alive cells by 91% (*P* < 0.01) and ginsenoside Rg1 increased by 86% (*P* < 0.05) compared with A*β* treated alone group. For MAP2 staining, Gp treated group had stronger signal than A*β* treated group, but Rg1 group had not (Figure 1(c)). The concentrations of geniposide and ginsenoside Rg1 were equivalent to the content in high concentration of TLJN. These results suggest that TLJN has efficacy on rescue of the primary cultured neurons in the presence of amyloid *β* because of geniposide and ginsenoside Rg1.

### 3.2. TLJN and Geniposide Promoted Neurites Outgrowth in A*β*-Insulted Rat Hippocampal Neurons

**Figure 2:**
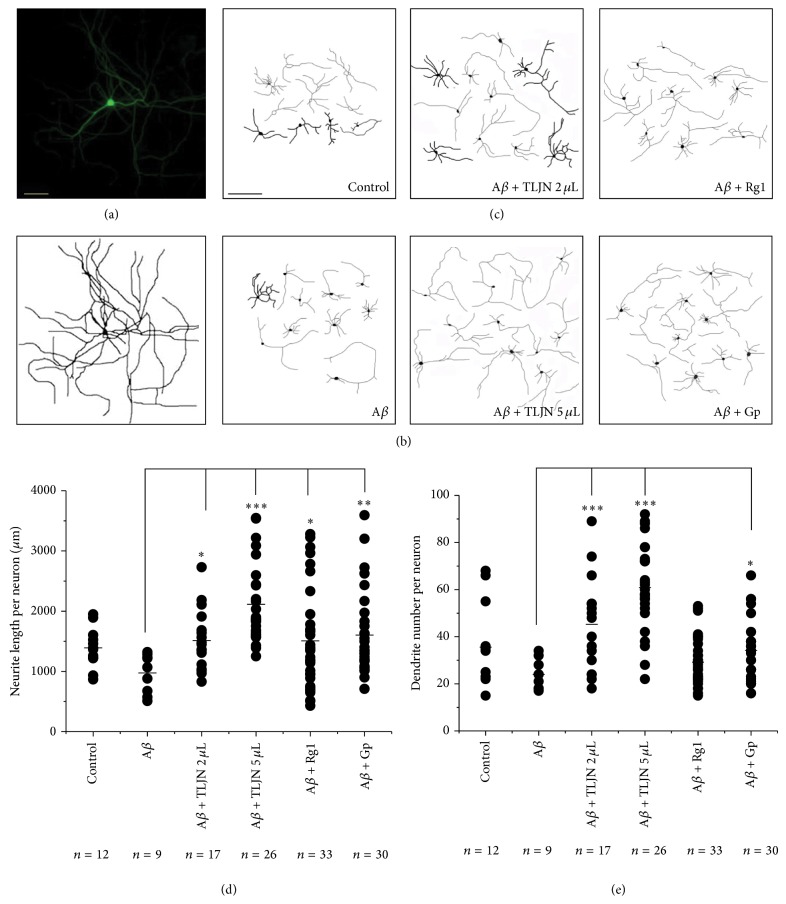
Effects of TLJN and its ingredients on neurite outgrowth of rat hippocampal neurons injured by A*β*. Rat hippocampal neurons were transfected with GFP for 3 days and observed under a fluorescence microscope (a) and its analog pictures were output (b, c). Bar = 25 *μ*m (a, b). Neurites of the GFP-positive neurons cultured with the mixture of TLJN (high dose of TLJN: 5 *μ*L TLJN per mL culture media (containing 83.5 *μ*M geniposide and 8.35 *μ*M ginsenoside Rg1); low dose of TLJN: 2 *μ*L TLJN per mL culture media (containing 25.4 *μ*M geniposide and 2.54 *μ*M ginsenoside Rg1)) or its ingredient (geniposide (83.5 *μ*M) and ginsenoside Rg1 (8.35 *μ*M)) and A*β* (10 *μ*M) were captured and drawn by using Image-Pro-Plus 5.0 software. Quantification of the length and number of neuritis of the neurons (d, e). Data were represented as mean ± standard deviation (SD); ^*^ *P* < 0.05, ^**^ *P* < 0.01, and ^***^ *P* < 0.001.

In above-mentioned experiments, we had observed that TLJN could decrease cytotoxicity of A*β*42 and so protect hippocampal neurons by inhibiting the cell death rate. To investigate whether TLJN or its ingredients could protect the neurons by promoting the outgrowth of neurites treated with A*β*42, we analyzed the neurites growth in rat hippocampal neurons coincubated with or without TLJN and its ingredients. The neurons were also transfected with GFP to facilitate measurements of neurites, and the length and number of processes of GFP-transfected neurons were measured with Image-Pro-Plus 5.0 software on the pictures taken by a fluorescent microscope (Figures 2(a), 2(b), and 2(c)).

Our results indicated that TLJN and geniposide could promote the outgrowth of neurites both in their length and in the dendrite number per neuron. The neurites length per neuron in high dose group of TLJN was increased by 118% compared with A*β*42 cultured group (*P* < 0.001), while the low dose group of TLJN, the group of Rg1, or the group of Gp increases by 56% (*P* < 0.05), 55% (*P* < 0.05), and 65% (*P* < 0.01), respectively, in the length of neurites (Figure 2(d)). Similar results were observed in the number of branches per neuron, which were increased by 99% (*P* < 0.001) and 168% (*P* < 0.001), compared with control group in TLJN-low dose group and TLJN-high dose group, respectively (Figure 2(e)). Geniposide also promoted neurites outgrowth by 50% compared with A*β*42 cultured group (*P* < 0.05), but ginsenoside Rg1 had no significance in increasing the number of branches per neuron. DMSO, however, which acted as negative group had effect on neither the length nor the number of branches in the process of neurons growth. This indicates that geniposide as a main component plays a major role in neurites growth. Therefore, our study was focused on the effects of geniposide instead of TLJN.

### 3.3. Geniposide, the Main Component of TLJN, Increasing the Viability of SY5Y-APP695swe Cells

**Figure 3:**
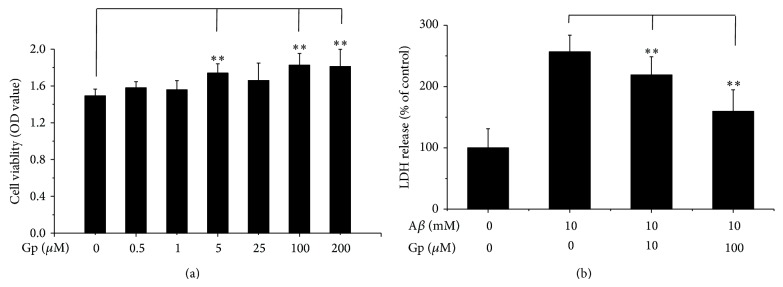
Geniposide increases the viability of SY5Y-APP695swe cells and decreases the cytotoxicity of A*β*. Different concentrations of geniposide (Gp) were added to swAPP-SY5Y cells. After 24 h coincubation, cell viability was detected by using CCK-8 Kit. Data were represented as mean ± standard deviation (SD), ^*^ *P* < 0.05, ^**^ *P* < 0.01 (a). Amyloid *β* was preincubated with geniposide (Gp) at 37°C for 24 h, and then the mixture was added to the hippocampal neurons. The concentration of LDH in medium was detected after incubation for 3 days (b).

To demonstrate that geniposide plays the major role in the protection of cells, SY5Y-APP695swe cell line was used as previously described [24]. To evaluate the protection effect of geniposide on SY5Y-APP695swe cells, they were treated with increasing concentrations of geniposide (0, 0.5, 1, 5, 25, 100, and 200 *μ*M) for 24 h (Figure 3(a)). Incubation with geniposide showed a dose-dependent increase in cell viability, with increasing cell viability by 22% at 100 *μ*M measured by CCK-8 assay.

To investigate whether geniposide can rescue the cells which suffered from A*β*42 aggregates, we determined the cytotoxicity of A*β*42 toward SH-SY5Y cells by detecting the LDH release in the medium after coincubation with different concentrations of geniposide at 37°C for 1 day. Coincubation of A*β*42 with geniposide showed a dose-dependent decrease in toxicity, with protection against A*β*42 toxicity at 100 *μ*M of geniposide by 45% and partial protection at 10 *μ*M by reducing LDH activity by 20% (Figure 3(b)). This indicates that geniposide is able to protect the cells under the treatment of A*β*42 aggregates.

### 3.4. Geniposide Stimulating High Molecular Mass Amyloidogenesis

**Figure 4:**
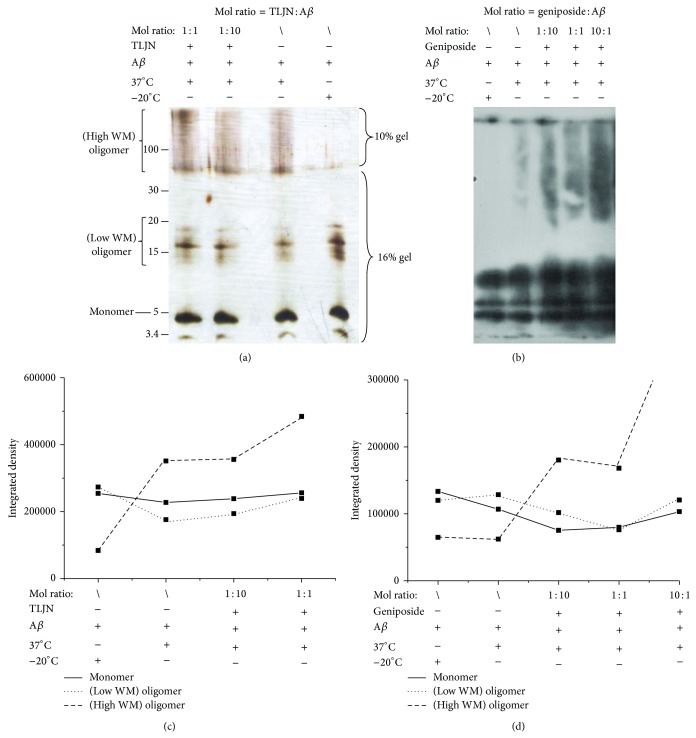
TLJN and geniposide influenced the aggregation of A*β*. After preincubation with TLJN or geniposide at different concentrations at 37°C for 24 h, the status of A*β* oligomers was measured with SDS-PAGE, silver staining (a, c), and Western blotting (b, d).

Now, we explore the putative mechanism of geniposide in its protection of neurons. First, to establish the influence of geniposide and TLJN with A*β* polymerization in aspect of molecular masses when polymers were formed, we monitored the peptide assembly using SDS-PAGE, silver staining (Figure 4(a)), and Western blotting (Figure 4(b)). High molecular masses of A*β* polymers were observed and smeared on the PAGE after the incubation at 37°C for 24 h. Geniposide increased the formation of high molecular mass A*β* polymers in dose-dependent manner which were retained in the 10% gel. More geniposide-generated A*β*42 polymers were presented as high molecular masses region in SDS stable structures that were retained in the gel. Similar effect could be seen in TLJN treated group as a positive control. Changes in gray density (Figures 4(c) and 4(d)) showed that both TLJN and geniposide increase high molecular mass A*β*42 polymers but do not decrease oligomers. These results suggest that geniposide promotes the formation of high molecular masses of A*β*42 polymers and thus decreases the quantity and cytotoxicity of A*β*42 oligomers.

**Figure 5:**
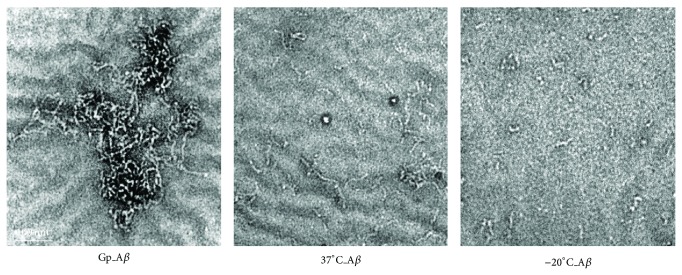
Geniposide promotes the formation of amyloid fibrils rather than protofibrils. A*β* (100 *μ*M) was coincubated with geniposide (100 *μ*M) at 37°C for 24 h, and the A*β* coincubated with PBS at 37°C or 4°C for 24 h was set as control. Morphology of the protein was observed by transmission electron microscope (scale bar represents 100 nm).

In order to demonstrate whether geniposide-induced polymers are fibrils, we incubated geniposide with A*β* and observed the samples with electron microcopy. As shown in Figure 5, A*β* fibrils were observed in the presence of geniposide. The geniposide-induced A*β* fibrils were more and longer than those in the absence of geniposide either at 37°C or at −20°C as controls. These results indicate that geniposide is able to promote fibrillogenesis and leads to a low cytotoxicity of A*β* polymers with high molecular masses.

**Figure 6:**
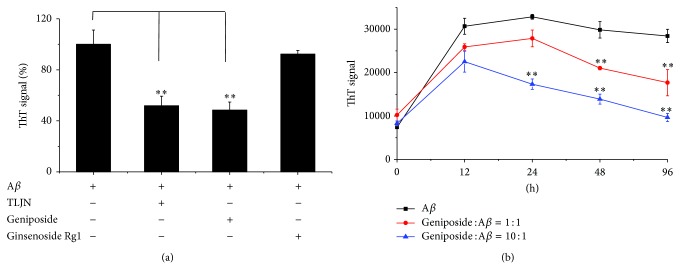
Preincubation of geniposide with A*β* inhibiting the formation of *β*-sheet in A*β*. Amyloid *β* was incubated with TLJN or its ingredients (geniposide∖ginsenoside Rg1) at 37°C for 24 h, and A*β* fibrillogenesis was measured with ThT fluorescence (450 nm) (a). Geniposide at different concentrations was incubated with A*β*, followed by measurement of the fluorescent intensity of ThT at different time intervals (b). Data were represented as mean ± standard deviation (SD), ^*^ *P* < 0.05, ^**^ *P* < 0.01.

To analyze the characteristics of geniposide on A*β* polymerization, we employed an experiment using thioflavin T, a commonly fluorescent dye (485 nm) binding to A*β* aggregates [25]. Amyloid *β* peptide was incubated in the presence and absence of TLJN as control groups (Figure 6(a)). Fibrillogenesis was also measured with the ThT fluorescence after incubation with ThT solution. In the absence of TLJN, we could measure the ThT-positive emission, *β*-sheet-rich aggregates after 24 h incubation. In contrast, formation of A*β* aggregates was suppressed in the presence of TLJN, in which TLJN reduced the ThT fluorescence to 49% (*P* < 0.01) compared with A*β* alone group. Sequentially, we examined the effects of geniposide and ginsenoside Rg1 on A*β* aggregations, separately. An equimolar concentration of geniposide relative to A*β* reduced the ThT fluorescence to 52% (*P* < 0.01), but ginsenoside Rg1 could not (*P* > 0.05).

Kinetics study of changes in ThT fluorescence showed that A*β*, when incubated alone, exhibited a time dependent increase in fluorescence. Coincubation of A*β* with geniposide inhibited amyloidogenesis of the peptide in a dose-dependent manner (Figure 6(b)). The extent of inhibiting aggregation was from 38% (molar ratio of geniposide: A*β* = 1 : 1) to 66% (molar ratio of geniposide: A*β* = 10 : 1) after 96 h treatment. That is to say, geniposide protecting and rescuing neurons may be resulting from its decrease in the formation of A*β* aggregates.

## 4. Discussion

Amyloid plaques and neurofibrillary tangles are two of the pathological hallmarks of AD. The major component of the amyloid plaques is A*β*. A key event in AD pathogenesis is the conversion of A*β* peptide from soluble monomer to aggregated forms in the brain [1, 2]. Preventing or reducing A*β* aggregation is one kind of promising therapeutic strategies under development or in clinical trials. Some papers reported that natural polyphenolic compounds could modify the pathway of A*β* aggregation, such as curcumin, EGCG, and ginkgo biloba [5, 11, 12]. Many works have examined the effects of polyphenols both on the direct interaction in the progress of A*β* fibrillogenesis and on secondary effects, such as A*β*-induced proapoptotic mechanisms [5, 11].

In our previous studies, we found that TLJN could upregulate levels of synaptophysin, NEP, and IDE in both the hippocampus and the cortex, protect neurons from A*β* toxicity, and promote the degradation of A*β* and clear amyloid plaque from the A*β* injection rat brains [15]. Meanwhile, TLJN could also influence amyloidogenic APP processing by downregulating the cleavage enzymes BACE1 and *γ*-secretase [16]. Besides, we also try to investigate the neuroprotection effect of TLJN in vitro and found that TLJN could attenuate oxygen and glucose deprivation induced neurotoxicity in primary rat hippocampal neuronal culture as well [23]. In order to explore whether TLJN could rescue neurotoxicity induced by A*β*, we used A*β*-injured primary rat hippocampal neurons as cell model and measured its cell viability and neuritis outgrowth after incubation with TLJN and its components. The results indicated that, coincubating with A*β* and TLJN, the LDH release was reduced and the survival rate was increased in hippocampal neurons compared to control. Besides, TLJN could promote neuritis outgrowth which also demonstrated that TLJN as well as its components could reverse the neurotoxicity of A*β*. So, we could confirm that both geniposide and ginsenoside Rg1, the two main components of TLJN, contribute to the neuroprotection effect of TLJN against A*β* while their mechanism was unclear.

Since the inhibition of A*β* aggregation is a potential target for protecting neurons from its toxicity, we detected whether TLJN could have influenced the aggregation of A*β*. The present study indicates that the ThT fluorescence in TLJN treated group was reduced by 49%, and this means that TLJN has an influence in the aggregation of A*β*, especially disturbing A*β* converting into a *β*-sheet conformation. As mentioned previously, geniposide and ginsenoside Rg1 are the main ingredients of TLJN. So, after confirming that TLJN could disturb A*β* aggregation, we detected which ingredient of TLJN could exert this effect. Our results showed that geniposide could reduce the ThT fluorescence to 52%, while ginsenoside Rg1 could not. Geniposide is an iridoid glycoside extracted from* Gardenia jasminoides Ellis* fruits. Geniposide has been shown to have antidiabetic, antiinflammatory, detoxifying, antioxidative, and antiangiogenic properties [26]. ThT is a benzothiazole salt obtained by the methylation of dehydrothiotoluidine with methanol in the presence of hydrochloric acid and is widely used to visualize and quantify the presence of misfolded protein aggregates called amyloid, both in vitro and in vivo [27]. When ThT binds to *β*-sheet-rich structures, such as those in amyloid aggregates, the dye displays enhanced fluorescence and a characteristic red shift of its emission spectrum [27]. TLJN and geniposide could decrease *β*-sheet structure in the process of A*β* aggregation in a dose-dependent manner. On the other hand, according to Cloe and colleagues [28], although thioflavin T fluorescence is often used as a diagnostic of amyloid structure, it is not perfectly specific for amyloid. Depending on the particular protein and experimental conditions, some amyloid fibers do not affect thioflavin T fluorescence raising the prospect of false negative results. It appears that geniposide-induced polymers may form different types of A*β* aggregation, which is not so sensitive to ThT measurement, compared with non-geniposide-induced A*β* polymers.

In order to study molecular weight when oligomers are formed, we monitored aggregate assembly using SDS-PAGE, silver staining, and Western blot. Silver staining shows that there were more high molecular weight SDS stable structures in TLJN treated group than A*β* alone group. Similar results could be seen in geniposide treated A*β*, and geniposide increased high molecular weight SDS stable structures that were retained in the gel with dose-dependent manner.

Electron microscope was used to observe the fibrillogenesis of A*β* incubated with geniposide. Longer and more fibril and much more condensed fibril knob can be seen when coincubating with geniposide. In our studies, geniposide may bind to a region in A*β* and stabilize intramolecular interactions in the protein. As we all know, the plaques composed of A*β* high mass polymers are less toxic compared to the soluble oligomer. It was pointed out that brain oligomeric *β*-amyloid but not total amyloid plaque burden correlates with neuronal loss and astrocyte inflammatory response in APP/tau transgenic mice [29]. Besides this, researchers found that amyloid *β* oligomers in aging and Alzheimer's disease and more related to the cognition impairment [30].

Combined with ThT fluorescence, SDS-PAGE, and EM experiment, we propose that geniposide may modulate the folding pathway of aggregation-prone polypeptides and thereby prevents their assembly into toxic aggregation products. Behrends and colleagues found that the chaperonin TRiC is a potent modulator of amyloid formation and stimulates the assembly of soluble polyglutamine-containing huntingtin oligomers of a new type which are nontoxic and are probably formed by an alternative aggregate formation pathway [31]. Ehrnhoefer et al.'s study showed that EGCG binds to natively unstructured A*β* monomers and prevents their conversion into stable, *β*-sheet-rich structures that acted as chemical chaperone [5]. Our study implies that geniposide may act with the same role like EGCG which redirects amyloidogenic molecules into off-pathway aggregation. Cell toxicity assay showed that the coincubation of A*β*42 with geniposide has a dose-dependent decrease in toxicity compared to A*β* alone group. So geniposide-generated off-pathway aggregation has less toxicity than normal aggregation. Geniposide could increase the cell activity of SY5Y-APP695sw cells with dose-dependent manner. The A*β* secreted by SY5Y-APP695sw cells in the culture medium could inhibit the activity of cells. Geniposide incubated together with SY5Y-APP695sw cells decreases the toxicity of secreted A*β*. Since the amyloid plaque is one of the early pathophysiological evidences in the long predementia phase of brain of the AD patients [32, 33], geniposide with the potential to accelerate less cytotoxic fibrillogenesis of A*β* may provide an opportunity for early treatment of AD.

## 5. Conclusion

**Figure 7:**
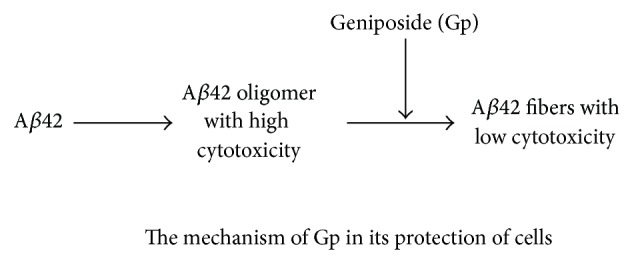
A putative mechanism for geniposide protecting and rescue neurons in the presence of A*β*.

On the basis of the studies presented here, we conclude that geniposide, the major component of TLJN, may have considerable potential as drug candidates for the treatment of neurodegeneration and amyloid diseases. The mechanism for geniposide to protect neural cells is resulting from its accelerating fibrillogenesis (Figure 7). Future studies will explore structural relationship with respect to the prevention of oligomerization and use cell and transgenic animal models of neurodegenerative diseases to elucidate the effect of geniposide on protein misfolding, aggregation, and toxicity in vivo.
